# Draft genome sequences of six *Brucella melitensis* isolates collected from humans and livestock in Tanzania

**DOI:** 10.1128/mra.01208-24

**Published:** 2025-05-23

**Authors:** Earl A. Middlebrook, Ephrasia Hugho, Beatus Lyimo, Coletha Mathew, Charles Mayenga, Lingling Li, Nelson B. Amani, AbdulHamid Lukambagire, Happiness Kumburu, Lidia Munuo, Samson Lyimo, Gabriel Shirima, Rudovick R. Kazwala, Maurice Byukusenge, Blandina Mmbaga, Zachariah Makondo, Vivek Kapur, Joram Buza, Jeanne M. Fair, Robab Katani

**Affiliations:** 1 Genomics and Bioanalytics Group, Los Alamos National Laboratory 5112, Los Alamos, New Mexico, USA; 2 Kilimanjaro Clinical Research Institute 668334, Moshi Urban, Kilimanjaro Region, Tanzania; 3 Kilimanjaro Christian Medical University College 108094 , Moshi, Tanzania; 4 Nelson Mandela African Institute of Science and Technology 248470, Arusha, Tanzania; 5 Sokoine University of Agriculture 108091, Morogoro, Tanzania; 6 Tanzania Veterinary Laboratory Agency 643316 , Dar es Salaam, Tanzania; 7 Department of Veterinary and Biomedical Sciences, The Pennsylvania State University 311374, University Park, Pennsylvania, USA; 8 Huck Institutes of the Life Sciences, Pennsylvania State University 124474, University Park, Pennsylvania, USA; 9 Department of Animal Science, Pennsylvania State University 227772, University Park, Pennsylvania, USA; The University of Arizona, Tucson, Arizona, USA

**Keywords:** zoonosis, biosurveillance, brucellosis, goat, livestock, spillover

## Abstract

We present genome assemblies of six *Brucella melitensis* strains isolated from goats and humans in Tanzania’s Kagera region. These sequences provide insight into circulating *Brucella* strains in Tanzania and East Africa. These data will support future comparative genomics, epidemiological investigations, and regional brucellosis control strategies.

## ANNOUNCEMENT

The molecular epidemiology of brucellosis in Tanzania is critical for effective disease control. The country’s heterogeneous agricultural practices create complex multi-host transmission networks for *Brucella* species. Whole genome sequencing and phylogenetic analyses comparing Tanzanian isolates with global data sets provide insights into strain diversity and regional pathogen dissemination patterns across East Africa (1).

*Brucella melitensis*, the predominant cause of human brucellosis, poses significant public health challenges on the African continent (2, 3). Here, we present six *Brucella melitensis* isolates from the Kagera region of Tanzania, providing molecular insights into strain diversity from this important livestock-producing area. Three isolates were obtained from goat milk (TZ_Kagera_G0-) and three from human patients in Karagwe district (TZ_Kagera_H0-). The animals and humans sampled were from a region where goats intermingled with cattle and sheep during grazing. Goat milk was collected (~5 mL) into sterile Falcon tubes, pooled from all four quarters after cleaning the udder and discarding the first few drops. Collected milk was immediately stored at 18°C. For human subjects, 5 mL of blood was aseptically taken by registered nurses from the brachial vein using heparinized tubes and immediately stored at −20°C. Both blood and milk were cultured on *Brucella* selective medium (Farrell’s medium) under both aerobic and anaerobic conditions (5% CO_2_) at 37°C for about 4 days (4, 5). Suspected Brucella colonies were subcultured to obtain pure isolates. Cultures were incubated at 37°C in 5%–10% CO_2_ and observed for 10 days. Suspected Brucella-positive cultures were confirmed by subculture on Farrell’s medium and MacConkey agar (6).

Isolates were plated on blood agar and incubated at 37°C in 5%–10% CO_2_ for up to 3 days. Two to three colonies were selected, heat-inactivated at 100°C for 10 minutes (7), and DNA was extracted using a DNeasy Blood and Tissue Kit (Qiagen, Hilden, Germany). DNA quality was confirmed via qPCR targeting the Brucella-specific IS711 sequence (8), NanoDrop spectrophotometer, and Qubit 4.0 fluorometer. Genomic libraries were prepared using the Illumina DNA Prep kit. One sample was sequenced with the P1 600-cycle kit (Kagera_G01), and the others with the P1 300-cycle kit (2 × 151 bp reads), all on an Illumina NextSeq 2000 platform.

Sequence analysis was performed within the EDGE bioinformatics UI platform (v2.4.0) (9). Reads were trimmed and filtered using *faQC* (v2.08) (10) with three bases clipped from each end and removing reads below 20 average quality and/or 50 bp length. Reads were assembled using *IDBA* (v1.1.1) (11) with options "--pre_correction --mink 31 --maxk 121 --step 20 --min_contig 200.” *CheckM* (v1.2.2) (12) was used to estimate completeness and contamination, while *Prokka* (v1.14.5) (13) was used to predict coding sequences (CDSs), tRNAs, and rRNAs (Table 1). Contigs less than 700 bp long were filtered out of the final assemblies.

**TABLE 1:** Genome assembly statistics, annotation results, and accession numbers

	Sample					
	TZ_Kagera_H01	TZ_Kagera_H02	TZ_Kagera_H03	TZ_Kagera_G01	TZ_Kagera_G02	TZ_Kagera_G03
Genome size	3,280,749	3,281,053	3,281,099	3,277,679	3,283,154	3,283,061
Contigs	38	38	37	236	37	37
Longest contig	609,536	609,552	609,536	80,579	609,570	609,586
N50	143,366	174,766	174,766	23,443	174,791	174,791
Mean contig length	86,335.50	86,343.50	88,678.35	13,888.47	88,733.89	88,731.37
GC_percent	0.57	0.57	0.57	0.57	0.57	0.57
CDS^*a*^	3,125	3,128	3,126	3,195	3,129	3,129
rRNA^*a*^	3	3	3	4	3	3
tRNA^*a*^	49	51	50	50	51	51
Coding density	0.87	0.87	0.87	0.87	0.87	0.87
Completeness	99.45	99.45	99.45	99.06	99.45	99.45
Contamination	0.67	0.67	0.67	1.79	0.67	0.67
NCBI BioSample	SAMN44407420	SAMN44407421	SAMN44407422	SAMN44407423	SAMN44407424	SAMN44407425
NCBI accessions	JBIQWR000000000	JBIQWQ000000000	JBIQWP000000000	JBIQWU000000000	JBIQWT000000000	JBIQWS000000000
SRA accession	SRR31174980	SRR31174979	SRR31174978	SRR31174977	SRR31174976	SRR31174975
Raw reads	3,302,222	3,294,052	3,219,745	13,196,957	3,211,131	3,081,903
Collection GPS	−1.6034498 31.141049	−1.6034498 31.141049	−1.5346324 31.1533194	−1.312585 31.20451	−1.312585 31.20451	−1.312585 31.20451

^*a*^ Indicates stats from Prokka annotations, all others from CheckM.

**Fig 1:**
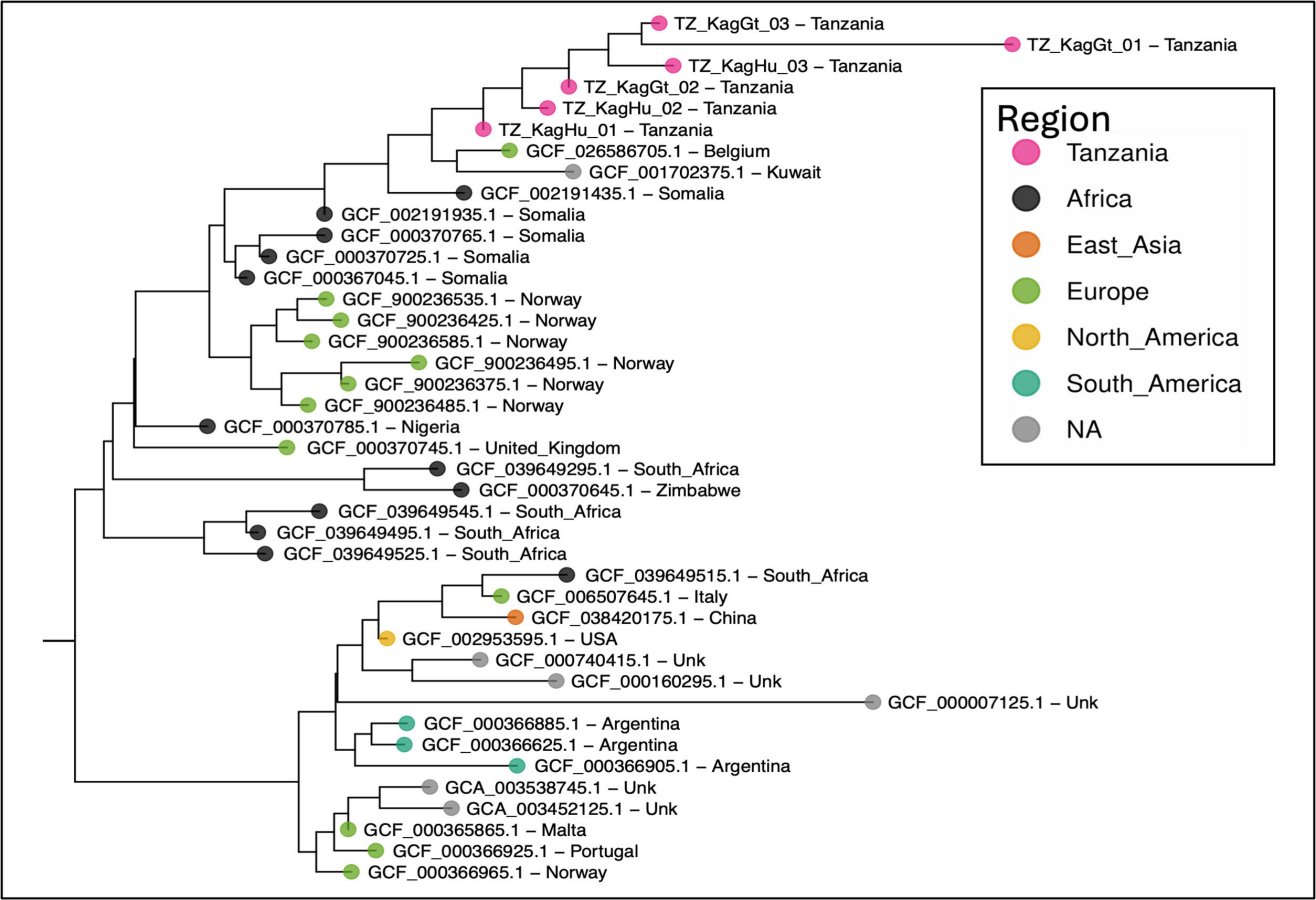
Phylogenomic tree of newly sequenced Brucella strains from Tanzania along with closely related sequences from NCBI assemblies. Leaves are labeled with NCBI’s accession and country of origin, and the symbol color at leaf tips indicates the broader region of origin, while the new sequences are indicated by pink symbols. Branch lengths are proportional to the inferred number of mutations/site. All splits are supported with 100% of aLRT 1000 replicates. Tree was inferred from all available *B. melitensis* assemblies in RefSeq/Genbank (accessed 3 Oct 2024) with the *OrthoPhyl* pipeline (v2.0) (14). OrthoPhyl was run with defaults except only strict single-copy orthologs were concatenated into the final codon supermatrix, and iqtree[] options “--lmap 2000 --symtest -B 1000 -t PARS --ninit 2 -m MFP -p partions.txt” were invoked. Tree was visualized with ggtree in R (15).

Phylogenetic relationship between the six new sequences and publicly available Brucella sequences shows the new sequences cluster tightly with the closest relatives being from Belgium, Kuwait, Somalia, and Norway (Fig. 1). These six new sequences increase our understanding of local *B. melitensis* strain diversity, supporting future comparative genomics, epidemiological studies, and regional brucellosis control efforts.

## Data Availability

These whole-genome projects have been deposited in NCBI’s GenBank under BioProject PRJNA1176626 and BioSample accessions SAMN44407420 to SAMN44407425. Raw reads have been deposited in NCBI’s SRA with accessions SRR31174975 to SRR31174980. Assemblies are available with accessions JBIQWP000000000 to JBIQWU000000000.

## References

[1] Ducrotoy MJ, Bertu WJ, Ocholi RA, Gusi AM, Bryssinckx W, Welburn S, Moriyón I. 2014. Brucellosis as an emerging threat in developing economies: lessons from Nigeria. PLoS Negl Trop Dis 8:e3008. doi:10.1371/journal.pntd.0003008 25058178 PMC4109902

[2] Georgi E, Walter MC, Pfalzgraf M-T, Northoff BH, Holdt LM, Scholz HC, Zoeller L, Zange S, Antwerpen MH. 2017. Whole genome sequencing of Brucella melitensis isolated from 57 patients in Germany reveals high diversity in strains from Middle East. PLoS One 12:e0175425. doi:10.1371/journal.pone.0175425 28388689 PMC5384748

[3] McDermott JJ, Arimi SM. 2002. Brucellosis in sub-Saharan Africa: epidemiology, control and impact. Vet Microbiol 90:111–134. doi:10.1016/s0378-1135(02)00249-3 12414138

[4] Farrell ID. 1974. The development of a new selective medium for the isolation of Brucella abortus from contaminated sources. Res Vet Sci 16:280–286. doi:10.1016/S0034-5288(18)33726-3 4369280

[5] Farrell ID, Robertson L. 1972. A comparison of various selective media, including a new selective medium for the isolation of brucellae from milk. J Appl Bacteriol 35:625–630. doi:10.1111/j.1365-2672.1972.tb03744.x 4631240

[6] Gopalsamy SN, Ramakrishnan A, Shariff MM, Gabel J, Brennan S, Drenzek C, Farley MM, Gaynes RP, Cartwright EJ. 2021. Brucellosis initially misidentified as Ochrobactrum anthropi bacteremia: a case report and review of the literature. Open Forum Infect Dis 8:ofab473. doi:10.1093/ofid/ofab473 34660837 PMC8514177

[7] Wakjira BS, Jorga E, Lakew M, Olani A, Tadesse B, Tuli G, Belaineh R, Abera S, Kinfe G, Gebre S. 2022. Animal brucellosis: seropositivity rates, isolation and molecular detection in southern and central Ethiopia. Vet Med (Auckl) 13:201–211. doi:10.2147/VMRR.S372455 36060523 PMC9431773

[8] Mancilla M, Ulloa M, López-Goñi I, Moriyón I, María Zárraga A. 2011. Identification of new IS711 insertion sites in Brucella abortus field isolates. BMC Microbiol 11:176. doi:10.1186/1471-2180-11-176 21813003 PMC3163539

[9] Li P-E, Lo C-C, Anderson JJ, Davenport KW, Bishop-Lilly KA, Xu Y, Ahmed S, Feng S, Mokashi VP, Chain PSG. 2017. Enabling the democratization of the genomics revolution with a fully integrated web-based bioinformatics platform. Nucleic Acids Res 45:67–80. doi:10.1093/nar/gkw1027 27899609 PMC5224473

[10] Lo C-C, Chain PSG. 2014. Rapid evaluation and quality control of next generation sequencing data with FaQCs. BMC Bioinformatics 15:366. doi:10.1186/s12859-014-0366-2 25408143 PMC4246454

[11] Peng Y, Leung HCM, Yiu SM, Chin FYL. 2012. IDBA-UD: a de novo assembler for single-cell and metagenomic sequencing data with highly uneven depth. Bioinformatics 28:1420–1428. doi:10.1093/bioinformatics/bts174 22495754

[12] Parks DH, Imelfort M, Skennerton CT, Hugenholtz P, Tyson GW. 2015. CheckM: assessing the quality of microbial genomes recovered from isolates, single cells, and metagenomes. Genome Res 25:1043–1055. doi:10.1101/gr.186072.114 25977477 PMC4484387

[13] Seemann T. 2014. Prokka: rapid prokaryotic genome annotation. Bioinformatics 30:2068–2069. doi:10.1093/bioinformatics/btu153 24642063

[14] Middlebrook EA, Katani R, Fair JM. 2024. OrthoPhyl-streamlining large-scale, orthology-based phylogenomic studies of bacteria at broad evolutionary scales. G3 (Bethesda) 14:jkae119. doi:10.1093/g3journal/jkae119 38839049 PMC11304591

[15] Yu G, Smith DK, Zhu H, Guan Y, Lam T-Y. 2017. ggtree: an R package for visualization and annotation of phylogenetic trees with their covariates and other associated data. Methods Ecol Evol 8:28–36. doi:10.1111/2041-210X.12628

